# Acoustic input and efferent activity regulate the expression of molecules involved in cochlear micromechanics

**DOI:** 10.3389/fnsys.2014.00253

**Published:** 2015-01-20

**Authors:** Veronica Lamas, Juan C. Arévalo, José M. Juiz, Miguel A. Merchán

**Affiliations:** ^1^Laboratory of Neurobiology of Hearing, Institute for Neuroscience of Castilla y Leon, University of SalamancaSalamanca, Spain; ^2^Facultad de Medicina de Albacete, Instituto de Investigación en Discapacidades Neurológicas (IDINE), Universidad de Castilla La ManchaAlbacete, Spain

**Keywords:** prestin oligomerization, acetylcholine α10 receptors, auditory cortex ablation, conductive hearing loss, descending control

## Abstract

Electromotile activity in auditory outer hair cells (OHCs) is essential for sound amplification. It relies on the highly specialized membrane motor protein prestin, and its interactions with the cytoskeleton. It is believed that the expression of prestin and related molecules involved in OHC electromotility may be dynamically regulated by signals from the acoustic environment. However little is known about the nature of such signals and how they affect the expression of molecules involved in electromotility in OHCs. We show evidence that prestin oligomerization is regulated, both at short and relatively long term, by acoustic input and descending efferent activity originating in the cortex, likely acting in concert. Unilateral removal of the middle ear ossicular chain reduces levels of trimeric prestin, particularly in the cochlea from the side of the lesion, whereas monomeric and dimeric forms are maintained or even increased in particular in the contralateral side, as shown in Western blots. Unilateral removal of the auditory cortex (AC), which likely causes an imbalance in descending efferent activity on the cochlea, also reduces levels of trimeric and tetrameric forms of prestin in the side ipsilateral to the lesion, whereas in the contralateral side prestin remains unaffected, or even increased in the case of trimeric and tetrameric forms. As far as efferent inputs are concerned, unilateral ablation of the AC up-regulates the expression of α10 nicotinic Ach receptor (nAChR) transcripts in the cochlea, as shown by RT-Quantitative real-time PCR (qPCR). This suggests that homeostatic synaptic scaling mechanisms may be involved in dynamically regulating OHC electromotility by medial olivocochlear efferents. Limited, unbalanced efferent activity after unilateral AC removal, also affects prestin and β-actin mRNA levels. These findings support that the concerted action of acoustic and efferent inputs to the cochlea is needed to regulate the expression of major molecules involved in OHC electromotility, both at the transcriptional and posttranscriptional levels.

## Introduction

Electromotility of outer hair cells (OHCs) in the organ of Corti is essential for active mechanical amplification of sound signals (Elgoyhen and Franchini, 2011). Somatic electromotility, i.e., the ability of OHCs to shorten or elongate in response to membrane voltage changes, depends on the unique properties of the membrane motor protein Prestin, whose voltage-dependent conformational changes are transferred to the actin cytoskeleton, a final effector of OHC micromechanical activation. The molecular structure of Prestin, its mechanisms and role in OHC electromotility, have been extensively studied (He et al., 2014). However, the complexity and extremely fast speed rates of operation of Prestin in particular and electromotility mechanisms in general, raise questions about regulation by incoming signals and possible adaptations to altered auditory input. There is some evidence that conductive hearing loss induces up-regulation of prestin mRNA (Mazurek et al., 2007; Yu et al., 2008; Yang et al., 2009). Up regulation of Prestin also has been reported after noise-induced hearing loss in preserved regions of the organ of Corti, consistent with compensatory mechanisms to stabilize thresholds and frequency discrimination (Xia et al., 2013).

Descending central feedback channeled through the efferent olivocochlear system is a major dynamic modulator of OHC electromotility and hence cochlear amplification (Elgoyhen and Franchini, 2011). Activity of medial olivocochlear cell (MOC) fibers acting on OHCs contributes to adapt cochlear gain by adjusting cochlear amplification. This effect is mediated by acetylcholine (Ach) released by MOC endings at the base of OHCs. Ach binds to a postsynaptic nicotinic Ach receptor (nAchR) assembled from α9 and α10 subunits whose activation hyperpolarizes OHCs. Ach-mediated hyperpolarization of OHCs has been linked to changes in axial electromotility amplitude and OHC compliance (He and Dallos, 1999, 2000).

Convergence of acoustic input and efferent connections on OHCs, raises the possibility that the expression of proteins involved in somatic electromotility is dynamically balanced by the interaction of signals from the acoustic environment and ongoing feedback from descending efferent inputs, primarily from MOC. To test this hypothesis, we compared the effects of a conductive unilateral hearing loss induced by removal of the ossicular chain (Sumner et al., 2005) with that of a partial deactivation of MOC activity by restricted ablation of the auditory cortex (AC). Previous work from our laboratory supports that in the rat AC restricted ablations induce a reversible deafness likely through descending, corticofugal control of MOC fibers (Lamas et al., 2013).

We tested changes in Prestin protein expression by Western blot after unilateral conductive hearing loss as a measure of regulation by acoustic input imbalance, and compared this with changes in Prestin protein expression after unilateral AC ablation. In parallel, because OHC electromotility regulation by MOC involves specialized cholinergic neurotransmission, we tested by qRT-PCR whether the expression of the alpha10 nAChR gene mRNA, a staple of cholinergic neurotransmission at the MOC-OHC synapse (Dallos et al., 1997; Maison et al., 2002, 2007; Batta et al., 2004; Vetter et al., 2007), changes along with the expression of Prestin and β-actin genes, as cell markers of the effects of MOC inactivation on OHC electromotility.

## Methods

This study was carried out in accordance with Spanish (Royal Decree 53/2013-Law 32/2007) and European Union (Directive 2010/63/EU) regulations on the care and use of animals in biomedical research.

Sixty eight young male Wistar rats weighing between 250–300 g were used in this study. One set of 12 animals was used in experiments of middle ear ossicle removal. Eight animals underwent surgical removal of the ossicular chain as described below, and were randomly assigned to one of two post-surgery day (PSD) survival groups, 7 PSD (*n* = 4) or 15 PSD (*n* = 4). The remaining animals were assigned to the normal control group (*n* = 4). For the cortical ablation experiments, a total of 49 animals were used. Forty-two underwent AC surgical removal, and were randomly assigned to one of three survival time groups: 1, 7 or 15 PSD (*n* = 14 for each PSD). The remaining 14 animals were used as normal controls.

### Surgical procedures

The AC ablations were performed under deep anesthesia using a mixture of ketamine chlorhydrate (30 mg/kg Imalgene 1000, Rhone Méreuse, Lyon, France) and xylazine chlorhydrate (5 mg/Kg, Rompun, Bayer, Leverkusen, Germany), as previously described in Lamas et al. (2013). The animals were returned to their cages after the ablations, carefully monitoring post-surgery recovery. Once the corresponding lesion survival time was completed, animals were anesthetized with 0.1 ml of sodium Pentobarbitol injected intraperitoneally (ip), and decapitated in order to collect the brain and the cochleae. After this, brains were immersed in 4% p-formaldehyde whereas the cochleae were immediately frozen in liquid nitrogen for qRT-PCR or Western blotting, as described below in detail.

The middle ear ossicular chain removal was performed under the same anesthetic cocktail and conditions. The left external acoustic meatus was exposed under microscopy control. The eardrum was punctured with the aid of a needle and the ossicular chain removed by extraction with tweezers. After surgery, the presence of an intact footplate attached to the oval window was confirmed under the microscope. Animals were returned to their cages after the ablations, carefully monitoring the post-surgery recovery. Once the corresponding post lesional survival times were completed, animals were anesthetized with 0.1 ml sodium Pentobarbitol, ip, and decapitated in order to collect the cochleae.

Once collected the cochleae were immediately frozen in liquid nitrogen for Western blotting.

### RNA extraction

To study expression of target mRNAs with qRT-PCR, total RNA was purified from the collected and homogenized cochleae using TRIZOL® (Gibco BRL, Gaithersburg, MD, USA) following the manufacturer’s protocol and a column from an RNeasy mini kit (Qiagen, Valencia, CA, USA) according to manufacturer’s instructions. RNA concentrations were determined using a NanoDrop ND-1000 spectrophotometer (NanoDrop Technologies Inc., Wilmington, USA). Each RNA sample was assayed three times and an average value was determined. RNA quality was assessed on an RNA 6000 NanoLabChip (Agilent Technologies, Palo Alto, CA, USA), using an Agilent 2100 Bioanalyzer to assess the integrity of the 18S and 28S rRNA bands, and an RNA integrity number (RIN) was assigned, with 0 corresponding to fully degraded RNA and 10 corresponding to intact RNA. For all Quantitative real-time PCR (qPCR), only RNA samples with RIN of at least 7.5 were used, with the vast majority of samples having a RIN of at least 8.0. These values meet requirements for reproductible qPCR experiments (Fleige et al., 2006).

### Quantitave real-time PCR

RNA (2 µg), primed with oligo-dT, was reverse-transcribed into cDNA at 37°C for 2 h using the first-strand cDNA synthesis kit (Promega Corporation, Madison, WI, USA) in a 20 µl volume, and stored at −20°C until use, according to manufacturer’s instructions. In all cases, a reverse transcriptase negative control was used for testing genomic DNA contamination.

qPCR was performed using the SYBR-Green method with a 2 × Master Mix (Applied Biosystems). Each reaction contained 10 µl of Master Mix, 0.4 µl of each pair of primers (Table 1), 3 µl of each cDNA sample in a different serial cDNA quantity for each gene, and MilliQ-grade water up to 20 µl. The amplification reaction took place in an ABI Prism 7000 detection system (Applied Biosystems), with the following conditions: 10 min at 95°C followed by 40 cycles of 15 s at 95°C and 1 min at 60°C depending on each pair of primers. Three PCR reactions were performed for each sample per plate, and each experiment was repeated twice. The ribosomal protein L-19 endogenous gene was used as reference gene.

**Table 1:** **Primers used in the RT-qPCR study**.

Gen	GenBank number	Primer forward	cDNA forward*	Primer reverse	cDNA reverse*	Product size	Slope	E**	R^2^
**b-act**	NM_031144	AGCCATGTACGTAGCCATCC	468–488	ACCCTCATAGATGGGCACAG	563–582	115	−3.15	107.4	0.996
**Chrna10**	NM_022639	CCTCACCTATGGCTGCTGCT	702–721	GCCAGCAGGGAGATGAACAC	805–824	123	−3.03	113.8	0.993
**Prestin**	NM_030840	GATTGGAGGTGTGGCCTGTCC	429–448	ACGGACATGGCGACTTTGAC	526–545	117	−3.11	109.6	0.995

*Location of the primers for the rat sequency in the Gene Bank. **Amplification efficiency.

The comparative threshold cycle (Ct) method was used to collect quantitative data (Schmittgen and Livak, 2008). Following the removal of outliers, raw fluorescence data were used to determine the PCR amplification efficiency (E) according to the formula E = [10^(−1/slope)^−1]^*^100. All amplifications had an E value of 100 ± 10%, the E value close to 100% being an indicator of efficient amplification. The relative gene expression value (“fold change”) for each transcript was calculated according to the equation E^−(ΔCt “condition 1”−ΔCt “condition 2”)^, where “condition 1” corresponds to experimental samples (PSD1, 7 and 15), “condition 2” to samples of control animals and ΔCt of each “condition” is Ct_“experimental gene”−_Ct_“endogenous gene”_ (Livak and Schmittgen, 2001; Schmittgen and Livak, 2008). A standard error for each relative gene expression value was calculated as a measure of data variation.

### Western-blot

Western blot analyses were performed according to Yu et al. (2011) with slight modifications. The cochleae were lysed in a lysis buffer (5 mM Tris pH 6.8, 2% SDS, 2 mM EDTA, 2 mM EGTA, 1 mM phenylmethylsulfonyl fluoride, 1 µg/mL aprotinin, 2 µg/mL leueptine, 1 mM vanadate, 10 mM sodium fluoride and 20 mM β-glycerophosphate) for 1 h at 4°C with gentle shaking and then centrifuged at 13000 × g for 15 min to eliminate debris. Protein concentration was quantified using Qubit Fluorometric quantification (Life Technologies). Lysates were added 5x SDS buffer and boiled for 6 min to denature the proteins. Proteins (20 µg/sample) were resolved by 7.5% SDS–PAGE and Western blots were performed with antibodies against the different proteins. Blots were developed in WesternBrigth ECL detection kit (Advansta). Films were digitized using an Epson V750 scanner.

Antibodies used were anti-Prestin (1:500) made in rabbit, a kind gift from Dr. Bechara Kachar (Laboratory of Cell Structure and Dynamics, National Institute on Deafness and Other Communication Disorders, NIH, Bethesda, Maryland), anti-beta tubulin (1:4000) and anti-actin (1:4000) from Sigma.

### ABR recordings

ABRs were recorded immediately before and after removal of the ossicular chain, and 7 days post injury. Recordings were performed using a close-field real-time signal processing system (Tucker-Davis Technologies (TDT), System 3, Alachua, Fl, USA), as previously described in Lamas et al. (2013).

**Figure 1:**
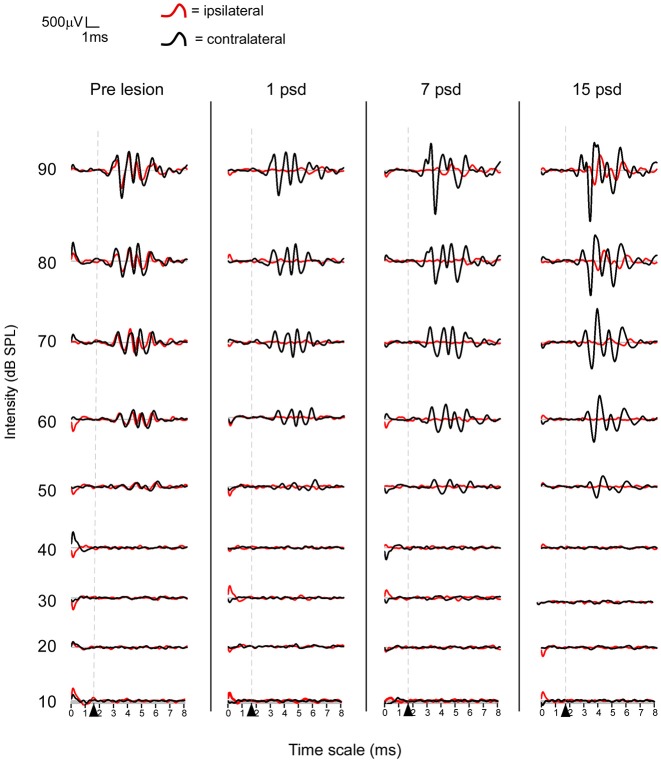
**Average ABR waveform obtained before and after the unilateral removal of the ossicular chain at different post surgery days (PSD), 1, 7 and 15**. The stimulus onset starts at 1.7 ms and all waves were visible at 50 dB SPL in both ears before the lesion. Note that only waves from the contralateral ear to the surgery remain visible at 50 dB SPL after the lesion.

On the side of the ossicle chain removal, the four characteristic waves of the rat ABR disappeared, thresholds were increased up to 90 dB SPL and did not show any recovery 7 days after the surgery (Figure 1). Fifteen days after the injury, waves were visible at 80 dB SPL. The side contralateral to the experimental lesion showed ABR thresholds similar to those of pre-lesion condition at all post lesional times (Figure 1). The amplitude of the four waves for this side was increased by 50–100% relative to pre-lesion condition at 15 PSD.

Changes in the ABR from animals with unilateral ablations of the AC have been previously described in Lamas et al. (2013). Briefly, on the side of the lesion the amplitude of the waves was decreased by 25–40% relative to pre-lesion condition at 1 PSD, and recovered at 7 PSD. The side contralateral to the ablation showed ABR amplitudes similar to those of pre-lesion condition at all post-lesion times.

### Statistical analysis

Real-time PCR results are shown as mean ± SD and were tested for significance using one-way ANOVA with Scheffe and Bonferroni *post hoc* tests. Ipsi- vs. contralateral statistical comparisons were carried out with Student’s *t*-test. Differences were considered significant at the *p* < 0.05 level. Statistical analysis was performed using the SPSS-IBM software, version 20 (SPSS Inc., Chicago, IL, USA).

### Localization of the lesions in AC

The localization of the lesions in AC was performed as described in Lamas et al. (2013). Briefly, after perfusion fixation and brain removal, the lateral surface of the brain was photographed using a Nikon camera located 21 cm above the cortex surface, and the photograph was superimposed to a purposely built coordinates map (Lamas et al., 2013). The extension of the lesion expressed in percent area of AC was calculated using the “area dimensioning tool” of Canvas X software (Lamas et al., 2013).

All ablations specifically encroached the major subdivisions of the AC (primary, dorsal and ventral cortices), and affected all AC layers but not the underlaying white matter. Lesions included a region ranging from 70 to 100% of the total AC area (Table 2).

**Table 2:** **Extent of lesions in the rat brain AC**.

Post surgery days	Percentage of AC ablated (WB study)	Percentage of AC ablated (RT-qPCR study)
1	73.33 ± 20.12	71.79 ± 8.33
7	75.73 ± 6.89	76.81 ± 9.24
15	81.79 ± 11.09	72.99 ± 5.27

*This table shows the percentage of AC affected by lesions in the experimental groups. Data are presented by Mean ± standard deviation. WB = Western Blott. RT-qPCR = Reverse Transcription quantitative polymerase chain reaction*.

## Results

### Prestin protein oligomerization after sound attenuation by unilateral middle ear ossicle removal

**Figure 2:**
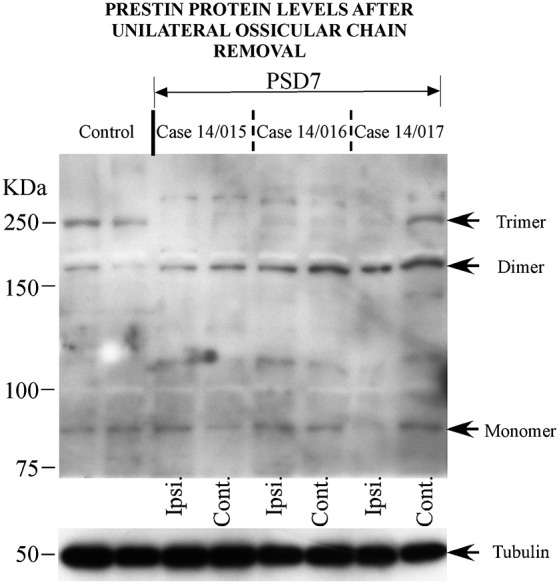
**Changes in prestin protein expression after unilateral removal of the ossicular chain**. Results from the ipsilateral (Ipsi.) and contralateral (Cont.) cochleae from three animals at 7 days after surgery (PSD7). The two tracks on the left are from two normal control animals.

Western blotting was performed to assess oligomerization and relative amount of Prestin protein in the cochlea at two survival times (7 or 15 PSD) after unilateral removal of the middle ear ossicles. Protein levels in the sides ipsi- and contralateral to the lesion were compared. After blotting cochlear samples with antiprestin antibodies, Prestin was displayed in Western blots from control animals in bands corresponding mostly to 80, 160 and 240 kDa (Figure 2—7 PSD, control animals on the two left tracks), in accordance with the reported molecular weights of the monomers, dimers and trimers of this protein, respectively (Matsuda et al., 2004).

There were no major differences in the pattern of immunoblot staining between both survival groups after middle ear ossicle removal. Figure 2 shows Western blots from three representative cases at 7 PSD. Western blots from animals at 15 PSD are not shown. Bands corresponding to Prestin monomers in animals after ossicle removal were comparable to those seen in control animals, regardless of survival time (Figure 2, lower arrow). However, the bands corresponding to prestin dimers (160 kDa) stood out more strongly labeled in animals with unilateral conductive deafness. This 160 kDa band was more marked in the side contralateral to the lesioned ear (Figure 2, second arrow from bottom). In general, bands corresponding to trimers were attenuated or absent at 7 and 15 PSD both in the cochleae ipsi- and contralateral to the lesion. Interestingly, however, in one case at 7 PSD after unilateral ossicle chain removal, the band corresponding to prestin trimers in the cochlea contralateral to the lesion, was comparable in intensity to that of controls (Figure 2, third arrow from the bottom).

Bands corresponding to the potential localization of tetramers (320 kDa) were faint or undetectable either in controls or in the conductive deafness experimental group at any survival time.

### Prestin protein oligomerization after attenuation of descending input to the cochlea by unilateral AC ablation

**Figure 3:**
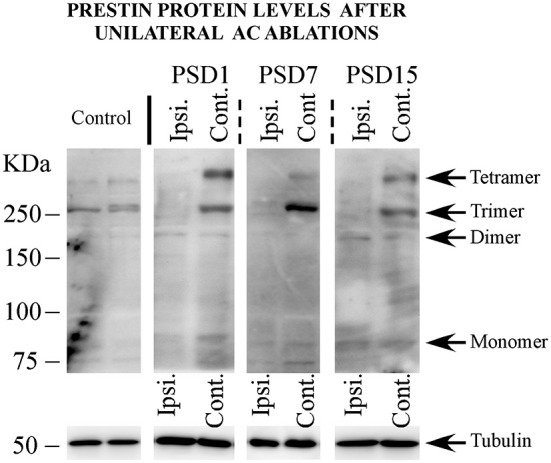
**Changes in the protein expression of prestin after unilateral ablation of AC at different post surgery days (PSD)**. Data represent the western blott results from both ipsilateral and contralateral ears to the surgery obtained at PSD1, 7 and 15. Ipsi = ipsilateral ear to the surgery. Contr. = contralateral ear to the surgery.

The effects of unilateral AC ablation on the oligomerization and relative amount of prestin protein in the cochlea, were also tested by Western blot. In the cochleae ipsilateral to the AC lesion, there were no immunolabeled bands above 160 KDa, indicating disappearance of trimeric Prestin forms relative to controls, with tetrameric forms being absent, like in control animals. Bands corresponding to monomers and dimers were unchanged relative to controls (Figure 3).

Western blot analysis of the cochleae contralateral to the AC lesion showed a banding pattern for monomers and dimers similar to that found in the ipsilateral side and therefore comparable to control cochleae. However, different to the ipsilateral side, dense bands at the 240 kDa location, corresponding to an intense expression of Prestin trimers were detected in most animals (Figure 3) at all survival times. A band at 320 kDa corresponding to Prestin tetramers was also visible in the side contralateral to the lesion.

No major differences were seen in the patterns of Prestin expression detected by Western blot at any of the three tested survival times, 1, 7 or 15 PSD (Figure 3).

### nAchR α10-subunit, Prestin and beta-actin mRNA levels after partial inactivation of descending input to the cochlea by AC ablation

We carried out qRT-PCR to assess changes in the expression of the α10-subunit of the OHC nAChR, Prestin and β-actin mRNA in the cochlea ipsi and contralateral to the lesion at different times after AC ablations. Collectively, these three markers should give an indication of how partial inactivation of MOC affects the molecular machinery of the OHC involved in electromotility.

**Figure 4:**
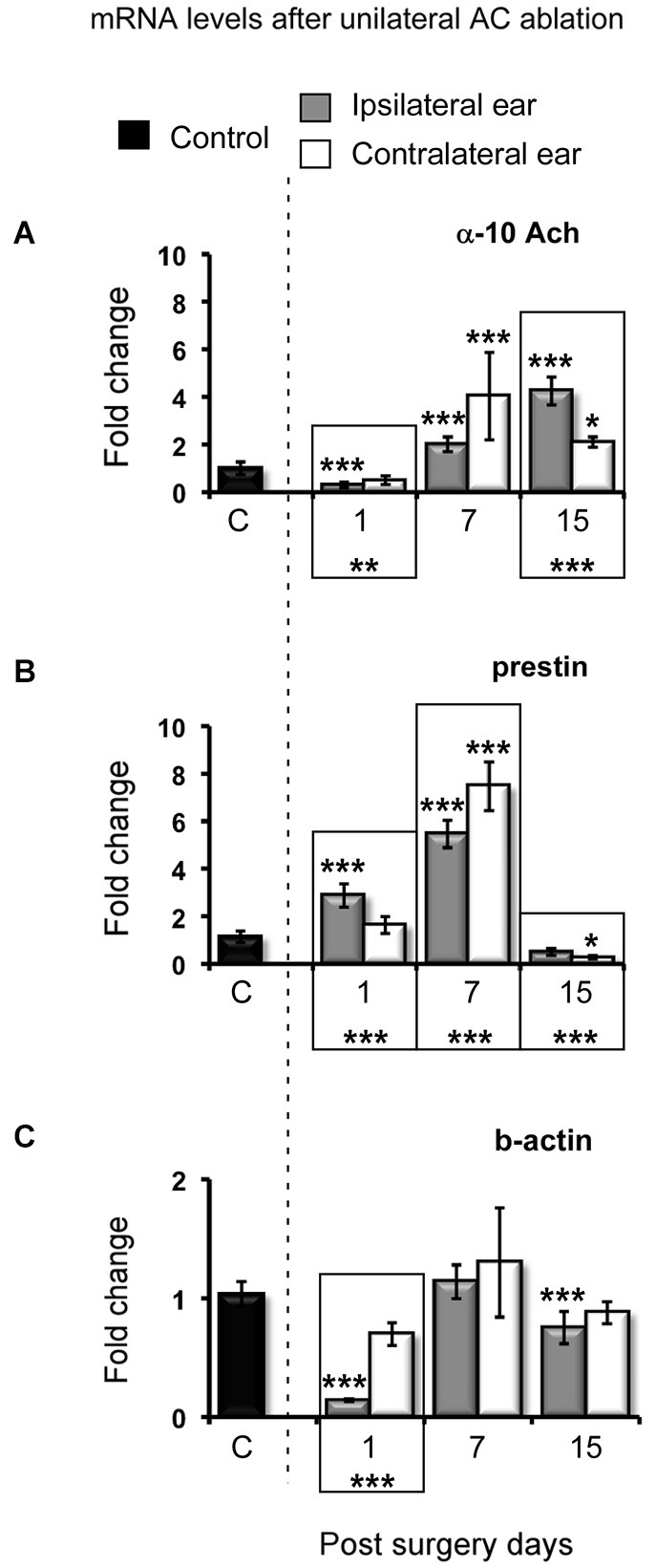
**Changes in the mRNA levels of α-10 subunit, prestin and β-actin after unilateral AC ablations at different post surgery days, 1, 7 and 15**. **(A)** Data of the *α-10 subunit* from the outer hair cell cholinergic receptor. **(B)** Data of *prestin*. **(C)** Data of β*-actin*. Results are presented by the mean ± stdev of the fold change. The statistical significance of the comparison between the fold changes of the post-surgery days and control condition is shown at the top of the bars. Bars from the same survival time that present differences between the ipsi- and contralateral ears are framed in a box, and their statistical significance is shown at the bottom. *p < 0.05; **p < 0.01; ***p < 0.001.

The α10-subunit transcripts of the nAChR showed a significant decrease in the cochleae ipsilateral to the lesion at 1 PSD after the AC ablation (Figure 4A). However, both at 7 and 15 PSD, α10 mRNA levels were significantly increased (Figure 4A gray bars). The increase in the α10-subunit transcripts was above two fold relative to control values at 7 PSD and above four fold at 15 PSD (Figure 4A).

The cochleae contralateral to the lesion did not show statistically significant changes in the expression of the α10 subunit at 1 PSD. However, both at 7 PSD and 15 PSD α10 mRNA levels were increased. These increases were four fold relative to control values at 7 PSD, and two fold at 15 PSD, respectively (Figure 4A white bars).

Prestin transcripts showed a significant threefold increase relative to control values in the cochlea ipsilateral to the lesion, at 1 PSD (Figure 4B gray bar). Increased transcript levels were even larger at 7 PSD, reaching six fold relative to controls, and returned to values similar to those of controls at 15 PSD (Figure 4B gray bars).

The cochlea contralateral to the lesion did not show any significant change in the expression of prestin at 1 PSD. An eight fold increase relative to control values was found at 7 PSD whereas at 15 PSD there was a significant decrease below control values (Figure 4B white bars).

The analysis of β-actin transcripts showed a significant decrease relative to control values in the cochlea ipsilateral to the lesion at 1 PSD following AC ablation. β-actin transcript levels returned to values comparable to those of controls at 7 PSD and decreased again at 15 PSD (*p* < 0.001) (Figure 4C gray bars). No statistically significant changes were found in the expression of the β-actin gene in the contralateral cochlea at any PSD after AC ablation (Figure 4C white bars).

## Discussion

The first key finding reported in this paper is that oligomerization of the OHC membrane motor protein Prestin is regulated by the interaction of acoustic input and centrifugal efferent cholinergic neurotransmission on OHCs.

In the normally functioning auditory receptor, monomeric and oligomeric forms of Prestin coexist, as shown in Western blots from control animals (see Figures 2, 3). Prestin oligomers include mostly dimers and trimers, whereas tetrameric forms are barely detectable in cochlear homogenates from control animals. The absence of tetrameric forms in our control Western blots is compatible with current views of Prestin oligomerization. Whereas tetramers may actually be present but not detectable in our control cochleae, the view that prestin functions strictly as a tetramer (Hallworth and Nichols, 2012) has been seriously challenged. Correlative electrophysiological and dynamic fluorescence measurements suggest that sub-tetrameric oligomers, or even monomers, are functional (Bian et al., 2013) and not mere precursors for the assembly of tetramers.

Dampened acoustic input subsequent to unilateral middle ear ossicle removal, diminishes prestin trimers, often below detection levels, in Western blots from cochleae in the side of the lesion. Dimeric forms, however, are preserved or even increased. These findings suggest that Prestin oligomerization beyond dimers requires normal acoustic input. Actually, Xia et al. (2013) have reported global increases in Prestin protein levels in the cochlea after noise-induced hearing loss, but these authors did not provide evidence as to whether such an increase was attributable to monomers or one or several oligomeric forms. The mechanism and role of this activity-dependent oligomerization will have to be elucidated. Shutting off oligomerization of larger Prestin forms while simultaneously increasing dimeric forms, might represent a mechanism to adapt cochlear micromechanics and amplification to diminished acoustic stimuli. On the other hand, the finding of relatively more dense dimeric bands in the cochlea contralateral to the lesion along with less frequent loss of trimers, may be related to subtle compensatory changes in the unmanipulated cochlea, driven by acoustic input imbalance between both ears, as discussed further in detail.

A second most relevant finding is that activity of the cochlear efferent system is also required to regulate Prestin oligomeric assembly. Evidence comes from results of ablation of the AC. Descending projections are complex, and include direct corticopontine and indirect colliculopontine pathways. At least corticopontine projections, are bilateral and symmetric, although more dense in the ipsilateral side (Doucet et al., 2002; Doucet and Ryugo, 2003; Schofield and Coomes, 2005), and innervate MOC in the ventral nucleus of the trapezoid body (Mulders and Robertson, 2000). MOC, in turn, send efferent axons bilaterally to OHCs, modulating its micro-mechanical properties (Guinan, 2006). Due to the excitatory nature of the corticofugal projection (Feliciano and Potashner, 1995), its degeneration after unilateral AC ablation induces loss of excitation on MOC, more marked in the ipsilateral side and, therefore an imbalance of efferent input between both ears (León et al., 2012).

After unilateral ablation of the AC, Prestin trimers were barely detectable in the cochlea ipsilateral to the lesion, whereas monomer and dimer bands were generally comparable to controls. In contrast, dense trimer bands were visible in the cochlea contralateral to the AC ablation. Prestin tetramers were also clearly detectable in the cochlea contralateral to the lesion. These findings suggest that diminished efferent activity on OHCs in the side of the AC ablation, also limits the assembly of higher forms of Prestin oligomers. Thus, monomeric and dimeric forms of Prestin might represent stable pools, relatively unaffected by changes in the acoustic or efferent input to the cochlea, at least after manipulations like those reported here. It is relevant that in the cochlea contralateral to the AC lesion, less affected by loss of efferent activity, Prestin trimers and tetramers are more intensely expressed. This may represent a mechanism to adapt cochlear amplification to efferent imbalance between both ears, whose intrinsic molecular nature is unknown.

Taken together, these findings support that Prestin oligomerization (Zheng et al., 2006; Mio et al., 2008) is regulated both by acoustic input and efferent activity. This may be part of mechanisms to adapt electromotility and cochlear amplification to altered inputs, including input imbalance between both ears. This prediction is worth pursuing through further experimental work.

A third finding is that efferent activity also regulates the expression of key genes involved in electromotility. The expression of the alpha10 subunit of the nAchR, Prestin and beta-actin genes are affected by diminished and unbalanced activity in the MOC, probably also as part of adaptations to altered input.

The main neurotransmitter in MOC is Ach (Warr, 1975; Altschuler et al., 1985; Vetter et al., 1991) which binds to an α9/α10 nAchR on OHCs (Elgoyhen et al., 1994, 2001), modulating motility and axial stiffness (Sziklai and Dallos, 1993; Sziklai et al., 1996, 2001; Dallos et al., 1997; Kalinec et al., 2000). In our unilateral AC ablation model, an expected loss of MOC cholinergic activity correlates with an acute (1 PSD) decrease in the gene expression of the α-10 subunit. This is followed by increased expression at 7 and 15 PSD, which is comparatively greater in the side ipsilateral to the AC lesion, where the activity of the descending corticopontine projection likely is less affected (Doucet et al., 2002; Doucet and Ryugo, 2003; Schofield and Coomes, 2005). This changes in the expression of the nAChR alpha-10 subunit point to OHC adaptation to loss of cholinergic input. The nature and function of such adaptations are unknown and need to be investigated in the future.

Likely as part of adaptive responses to limited MOC activity on OHCs, Prestin gene up regulation is seen in the cochlea ipsilateral to the AC ablation at short times (1 and 7 PSD) after the lesion, with a return to control values at 15 PSD. In the cochlea contralateral to the AC lesion, however, increased Prestin mRNA levels are found just at 7 PSD, with levels at 15 PSD slightly decreased relative to controls. Whereas these oscillations in Prestin gene expression do not bear a linear relation with changes in protein levels seen in Western blots, they probably represent additional levels of regulation by efferent activity or an indirect effect of GABA or CGRP release by the MOC terminals (Maison et al., 2003).

It is also interesting that there is a significant drop in the expression of the β-actin gene at 1 PSD in the cochlea ipsilateral to the cortical lesion. Down-regulation of the β-actin gene could be partly responsible of a greater motor deactivation (Matsumoto et al., 2010) and may reflect the effect on one of the final targets of efferent regulation on OHCs. This may be related to decreased activity in the ipsilateral cochlea, with higher thresholds and a decrease in the amplitude of ABR waves, after unilateral AC lesions (Lamas et al., 2013). In this regard it is interesting to note that at 15 days PSD, prestin gene expression returns to levels similar to controls, while expression levels of the nAChR α10 subunit are still increased. This change, which coincides in time with the recovery of the ABRs previously observed by us (Lamas et al., 2013), may reflect an OHC electromotile “resetting” induced by nAchr receptor adaptations at the MOC-OHC synapse. Therefore, the activity-dependent regulation of the α10 subunit, prestin and β-actin genes reported in this paper may reflect OHC adaptations to changes in MOC activity to compensate for limited and/or unbalanced corticofugal excitation.

In conclusion in this paper we suggest that at least two mechanisms are at work in combination to balance the micromechanical response of the OHC after a decrease in inner ear activity: changes in oligomerization of Prestin and MOC cholinergic neurotransmission along with regulation of the expression of Prestin and β-actin genes.

## Authors contributions

Miguel A. Merchán and Veronica Lamas designed the experiments. Veronica Lamas and Juan C. Arévalo performed the experiments and analyzed data. Veronica Lamas, Juan C. Arévalo, José M. Juiz and Miguel A. Merchán participated in the discussion of the results. José M. Juiz, Miguel A. Merchán and Veronica Lamas, wrote the paper.

## Conflict of interest statement

The authors declare that the research was conducted in the absence of any commercial or financial relationships that could be construed as a potential conflict of interest.
